# Cross-Species Spillover of Tiger Frog Virus Caused Lethal Systemic Disease in Captive *Geochelone sulcata*: Etiology, Pathology, and Genomic Characterization

**DOI:** 10.3390/v18080810

**Published:** 2026-07-23

**Authors:** Chuchu Lai, Fen Shan, Danning Song, Minxin Chen, Minghui He, Zujin Chen, Xuezhu Lee

**Affiliations:** 1 College of Marine Sciences, South China Agricultural University, Guangzhou 510642, China; 15976728306@163.com (C.L.); 19865992823@163.com (D.S.); 13268280623@163.com (M.C.); 15386042821@163.com (M.H.); 2 Guangzhou Zoo, Guangzhou 510070, China; shanfen_gzzoo@sina.com; 3 Guangzhou Wildlife Research Center, Guangzhou 510075, China; 4 Guangzhou Collaborative Innovation Center on Science-Tech of Ecology and Landscape, Guangzhou 510070, China

**Keywords:** *Geochelone sulcata*, Tiger Frog Virus, *Ranavirus*, cross-species transmission, metagenomics

## Abstract

In 2025, a severe mass mortality outbreak struck captive *Geochelone sulcata* at a zoo in Guangdong Province, China. To trace the causative agent, identify the viral strain and characterize associated pathological lesions, respiratory tract samples collected from diseased tortoises were subjected to a combined technical workflow. Metagenomic high-throughput sequencing was first applied to screen for potential pathogens, followed by virus isolation via cell culture. Transmission electron microscopy (TEM) was used to observe viral morphology. The major capsid protein (MCP) gene was amplified by PCR for molecular identification, and whole-genome sequencing together with phylogenetic analysis was performed to clarify the genetic features of the isolate. The results verified that ranavirus was the primary pathogen responsible for the mortality. Pathological examination demonstrated acute necrosis, hemorrhage and inflammatory infiltration in multiple organs including the liver, spleen, lung, and pancreas. TEM observation revealed typical iridovirus-like particles with an average diameter of approximately 70 nm. Molecular and genomic analyses confirmed the pathogen as Tiger Frog Virus (TFV) of the genus *Ranavirus*, designated TFV-CN2025, which shared 99.8% nucleotide sequence homology with known TFV reference strains. To our knowledge, this is the first report of lethal TFV infection in *G. sulcata*, which provides detailed pathological evidence for this cross-species transmission event from amphibian hosts to terrestrial chelonians. Our findings indicate that TFV poses considerable risks to the tortoise breeding industry and ecological security in China. We therefore suggest incorporating TFV detection into routine quarantine and disease surveillance programs for captive tortoises.

## 1. Introduction

In recent years, fueled by burgeoning domestic and international market demand, the tortoise aquaculture industry in Southern China has experienced rapid expansion [1]. The *Geochelone sulcata* belongs to the class Reptilia, order Testudinata, suborder Cryptodira, and family Testudinidae. As a representative terrestrial chelonian, the family Testudinidae comprises approximately 18 extant genera and 65 species globally, accounting for roughly 18% of the total diversity within the order Testudinata. This species exhibits robust environmental plasticity, thriving across diverse terrestrial ecosystems and evolving highly specialized niche strategies [2]. Owing to its significant economic value—with market prices exceeding ¥3000—and its utility in ecological education, *Geochelone sulcata* has been extensively introduced into captive breeding programs [1] and is frequently featured in zoological exhibitions and science popularization venues. However, the prevalence of high-density husbandry and deficient sanitary management in artificial environments renders these tortoises susceptible to pathogenic incursions, which poses great challenges to the healthy development of captive populations.

In 2025, a sudden mass mortality event occurred within a *Geochelone sulcata* population at a zoo in Guangdong Province, resulting in the sequential death of over twenty individuals. Given the high economic value of *G. sulcata*, traditional pathogen identification methods relying on microbial pure culture and artificial infection assays are difficult to implement in this species. To efficiently trace the causative agent, we adopted a combined strategy of metagenomic high-throughput sequencing and clinical virus isolation in this study. Upper respiratory tract samples collected from diseased tortoises were first subjected to metagenomic sequencing, and the results revealed a significant presence of viral sequences belonging to the family Iridoviridae. Further sequence comparison confirmed that the virus was a member of the genus *Ranavirus*. Subsequent virus isolation and identification verified that the pathogen was Tiger Frog Virus (TFV). Notably, this is the first report of TFV naturally infecting the terrestrial reptile *G. sulcata*, demonstrating a clear cross-species transmission event of TFV from its traditional amphibian hosts to land tortoises. This novel finding highlights the expanding host range of ranaviruses and reminds researchers to fully assess the host adaptability and potential ecological risks of such pathogens. Based on the above results, we further conducted systematic etiological research on this reptile-derived iridovirus.

Tiger Frog Virus (TFV) belongs to family *Iridoviridae*, genus *Ranavirus*, species *Ranavirus rana1*. It was historically recognized as a typical amphibian pathogen, predominantly infecting frogs and triggering numerous large-scale disease outbreaks in farmed and wild amphibian populations [3]. However, with the continuous advancement of molecular epidemiological investigations, studies have revealed that ranaviruses exhibit remarkable genetic diversity and evolutionary potential within natural environments, including an early documented *Ranavirus rana1*-related ranavirus strain capable of infecting soft-shelled turtles [4]. Kim [5] first identified endemic *Ranavirus rana1*-like ranaviruses in the wild Korean clawed salamander (*Onychodactylus koreanus*), confirming that these viruses have undergone profound phylogenetic divergence among wild populations in East Asia. Concurrently, Logan [6] detected variant strains in deceased wild wood frogs from Wood Buffalo National Park, Canada, which, despite sharing high homology with the prototype *Ranavirus rana1*, possess unique genomic signatures. The sustained genetic mutations and recombination events occurring within natural hosts furnish the fundamental molecular basis for the virus to breach preexisting ecological and interspecies barriers. Nevertheless, accumulating evidence in recent years indicates that ranaviruses possess an expansive host range; they are capable of infecting not only fish and amphibians but also reptiles and even invertebrates via cross-species transmission, thereby posing a severe threat to global biodiversity and ecological integrity [7]. Systematic collation of reptile ranavirus host distribution data has been published in a specialized chapter contributed by Marschang et al. [8] within the second edition of Ranaviruses: Emerging pathogens of ectothermic vertebrates. Meanwhile, outbreaks of ranavirus diseases have been frequently documented across the globe, including the United States, Canada, South Korea, China, the United Kingdom, Germany, France, Brazil, and Chile [9,10].

Although sporadic cases of ranavirus infection in atypical hosts have been reprted [11], systematic etiological investigations of reptile-derived ranaviruses remain scarce. In particular, the lethal pathogens and related viral strains associated with diseases in exotic terrestrial tortoises such as *G. sulcata* have not been clearly identified to date. As a valuable non-native species under protection, *G. sulcata* has been artificially bred at multiple bases across China [12]. These facilities not only carry out standardized ecological farming and wild population conservation work, but also provide favorable conditions for relevant scientific research and the preservation of the species’ gene pool. Previous studies on *G. sulcata* mainly focused on gonadal lesions and renal calculi, and there was also a published case report of cryptosporidiosis in this species [13]. However, viral infectious diseases of this tortoise have long been understudied.

The present study aimed to identify the specific viral strain responsible for the mass mortality of captive *G. sulcata*. The integrated application of metagenomic sequencing and virus isolation overcomes the limitations of traditional diagnostic techniques for high-value tortoise species, forming a reliable technical system for pathogen identification. Meanwhile, the discovery of TFV cross-species transmission to *G. sulcata* enriches the host spectrum of ranaviruses. The outcomes of this work will promote the inclusion of this pathogen into routine diagnostic screening for chelonian diseases, provide a scientific reference for disease prevention and control in captive breeding, and offer theoretical support and practical strategies for the health management of tortoise aquaculture and regional ecological security.

## 2. Materials and Methods

### 2.1. Animals and Cells

Upper respiratory tract tissue specimens from the afflicted *Geochelone sulcatas* were submitted for diagnostic examination by the breeding facility. Epithelioma Papulosum Cyprinid (EPC) cells is maintained by the College of Marine Sciences at South China Agricultural University.

### 2.2. Clinical Examination of Diseased Turtles

The clinical status of the symptomatic *Geochelone sulcatas* was documented via macroscopic observation. Integrating background data—including environmental parameters of the husbandry facility, anamnesis (medical history), pharmacological interventions, as well as morbidity and mortality rates—necropsies were performed under aseptic conditions. A systematic evaluation of the gross pathological features was conducted, supplemented by high-resolution digital photography for subsequent diagnostic analysis.

### 2.3. Metagenomic Profiling and Analysis of Symptomatic Geochelone sulcatas

The metagenomic sequencing service for the *Geochelone sulcatas* was provided by Wuhan Baiyi Huineng Biotechnology Co., Ltd. (Wuhan, China). Upper respiratory tract tissue samples were collected from the diseased *Geochelone sulcatas*, and total RNA extraction and quality control were performed on the samples. After obtaining qualified total RNA, ribosomal RNA (rRNA) was removed to enrich the target transcripts; the RNA fragments were randomly fragmented and screened for target-length fragments, which served as templates for the synthesis of the first-strand cDNA, followed by the synthesis of the second-strand cDNA to obtain double-stranded DNA fragments; end repair and 3′-“A” tailing were performed on the double-stranded DNA fragments, and sequencing adapters were ligated before the products were purified; the purified products underwent PCR amplification to construct the sequencing library. After the library passed quality testing, it was sequenced on a high-throughput sequencing platform to obtain raw sequence data for subsequent bioinformatics analysis.

After the total DNA of the samples passed the purity, concentration, and integrity tests, small-fragment libraries were prepared using the VAHTS Universal DNA Library Prep Kit for Illumina V3 (Vazyme Biotech, Nanjing, China). After fragmentation, adapter ligation, PCR amplification, and purification, the sequencing libraries were constructed; DNA nanoballs were prepared from the libraries via rolling circle amplification and subjected to PE150 paired-end sequencing on the DNBSEQ-T7 platform to obtain raw sequencing data.

The raw sequencing data were subjected to quality-control filtering and statistical processing using fastp (v0.21.0); if host sequences were present in the samples, the host sequences were removed first, and then kraken2 was employed to perform species annotation and abundance statistics at the reads level, combined with LEfSe and two-tailed *t*-tests to screen for differential species between groups. After genome assembly and result optimization of the quality-controlled sequences, ncRNA sequences were predicted and removed, followed by viral sequence prediction; species annotation, abundance analysis, and integrity assessment were carried out on the predicted viral sequences, and a non-redundant gene set was constructed to complete functional annotation through multi-database alignment. Community diversity analysis was conducted based on the gene set, while differential taxa between groups were simultaneously detected.

### 2.4. Histopathological Examination of Diseased Geochelone sulcatas

The lung, liver, spleen, trachea, kidney, intestine, pancreas, rectum, and testicular tissues were collected from the diseased *Geochelone sulcatas*, and immediately submerged in 10% neutral buffered formalin for 24 h to ensure adequate fixation. Following fixation, the tissues were rinsed twice with Phosphate-Buffered Saline (PBS) and subjected to a graded ethanol series (70%, 80%, 95%, and absolute ethanol) for dehydration, with each stage lasting one hour. The samples were subsequently cleared in xylene and embedded in paraffin wax.

Histological sections were prepared, mounted on glass slides, and incubated at 60 °C overnight. Hematoxylin and eosin (H&E) staining was performed as follows: sections were initially stained with hematoxylin for 5 min, differentiated in acid alcohol, and “blued” under running tap water; thereafter, the sections were counterstained with eosin for 3 min, followed by final dehydration, clearing, and mounting.

### 2.5. Virus Isolation

Liver, spleen, and kidney tissues were harvested from the diseased tortoises under aseptic conditions and homogenized in M199 culture medium supplemented with 2% fetal bovine serum (FBS), 100 U/mL penicillin, and 100 μg/mL streptomycin. The resulting homogenate underwent three consecutive freeze–thaw cycles at −80 °C to facilitate viral release, followed by centrifugation at 12,000× *g* for 30 min at 4 °C. The supernatant was collected as the crude viral suspension for subsequent use.

The filtered supernatant (1 mL) was inoculated onto EPC cells and incubated at 20 °C for 1 h to facilitate viral adsorption, following the universal receptor-dependent viral adsorption experimental framework validated across multiple rhabdovirus genera [14]. Subsequently, the inoculum was replaced with 5 mL M199 maintenance medium supplemented with 2% FBS, and the cultures were maintained under the same conditions. The cells were monitored daily for the development of Cytopathic Effect (CPE). Upon the manifestation of distinct CPE, the detached cells were gently harvested using a cell scraper. The cell suspension was then centrifuged according to the parameters, and the resulting supernatant was aliquoted and stored at −80 °C. The cell inoculation and virus release processing workflow referenced standardized operational specifications established for aquatic ranavirus in prior methodological research [15].

### 2.6. Ultrastructural Analysis of Viral Particles

The ultrastructural morphology of the viral particles was characterized utilizing TEM. The viral suspension obtained in Section 2.5 was subjected to a 2-fold serial dilution and subsequently inoculated into T25 flasks. The cultures were maintained in a constant-temperature incubator at 25 °C for 2–3 days. Upon the manifestation of a CPE involving 30–70% of the cell monolayer, the samples were harvested for electron microscopic preparation.

The culture supernatant was aspirated, and the adherent cells were recovered using a cell scraper and resuspended in 1 mL of M199 medium supplemented with 5% FBS. The resulting cell suspension was centrifuged at 1500 rpm for 10 min; following the removal of the supernatant, the pellet was initially stabilized with 0.5% glutaraldehyde at room temperature for 10 min. Subsequently, the sample was centrifuged at 10,000 rpm for 10 min at 4 °C, the supernatant discarded, and the pellet subjected to secondary fixation with 3% glutaraldehyde in the absence of light. Following secondary fixation, the sample was centrifuged at 10,000 rpm for 10 min at 4 °C, and the supernatant was carefully discarded. Dehydration, embedding, and ultramicrotomy were performed in accordance with standard histopathological protocols, followed by TEM examination to delineate the morphological attributes of the virions.

### 2.7. PCR Detection and Phylogenetic Analysis of Ranavirus

Liver, spleen, and kidney tissues collected from diseased tortoises were cut into rice-sized fragments. Total RNA was extracted using a genomic RNA extraction kit, (Foregene, Chengdu, China) followed by stringent quality assessment. The RNA was reverse-transcribed into cDNA utilizing TaKaRa reverse transcriptase (Takara Biotechnology (Dalian), Dalian, China) to serve as a template for subsequent PCR amplification. The specific PCR reaction system and thermocycling parameters are detailed in Table 1.

Primers were specifically designed based on the metagenomic sequencing results, targeting the MCP gene of the putative ranavirus. The primer sequences were as follows: forward, 5′-CACCTCCATCCCGACTCAGCA-3′; reverse, 5′-GAATCCCATGAGCCGTTCA-3′, with an expected amplicon size of 260 bp. These primers were synthesized by Tsingke Biotechnology Co., Ltd (Beijing, China). The thermal cycling conditions comprised an initial denaturation at 95 °C for 5 min, followed by 30 cycles of denaturation at 95 °C for 30 s, annealing at 57 °C for 30 s, and extension at 72 °C for 1 min, with a final elongation step at 72 °C for 10 min. ddH_2_O served as the negative control. Forward and reverse primers were stocked at 10 μM prior to setup. Commercial 2× rTaq premix was diluted to a standard 1× working concentration in the 25 μL reaction volume.

A 10 μL aliquot of the PCR products was subjected to 1% agarose gel electrophoresis and stained to verify the presence of the expected 260 bp bands. Positive amplicons were submitted to Tsingke Biotechnology Co., Ltd. for Sanger sequencing. Upon acquisition of the sequences, reference MCP gene sequences of other iridoviruses were retrieved from the NCBI database. Multiple sequence alignment and phylogenetic tree reconstruction were subsequently performed using MEGA 7.0 software.

### 2.8. TFV Whole-Genome Sequencing

Viral whole-genome sequencing services were provided by Wuhan Baiyi Huineng Biotechnology Co., Ltd. Following genomic DNA extraction, quality control was performed based on concentration, purity, and integrity. Qualified DNA underwent fragmentation and screening to obtain target-length fragments, which were then used to construct sequencing libraries through end repair, A-tailing, adapter ligation, and PCR amplification. After the libraries passed quality inspection, DNA nanoballs were prepared via denaturation, cyclization, and rolling circle amplification. These nanoballs were loaded onto microarray chips, and sequencing was completed on the DNBSEQ-T7 platform using cPAS technology to obtain raw sequencing data.

The raw data generated from sequencing were processed using fastp (v0.21.0) to filter out adapter sequences, low-quality reads, and sequences containing ambiguous bases, thereby yielding high-quality clean data. In instances where host sequence contamination was identified, viral sequences were selectively extracted prior to de novo genome assembly and polishing via unicycler (v0.4.8). SPAdes generate preliminary contig scaffolds with robust error tolerance, while Unicycler further optimizes scaffold connectivity and completes circularization suited for closed viral genome reconstruction. Post-assembly, the sequencing depth was quantified through alignments performed with minimap2 (v2.18-r1015), followed by circularization analysis and refinement of the assembly results. Subsequently, protein-coding genes were predicted using GeneMarkS. Functional annotations were executed across multiple specialized databases—including TIGRFAMs, Pfam, GO, KEGG, RefSeq, SwissProt, and COG—utilizing Interproscan (v5.30-69.0) and blastp. Finally, the structural and functional annotation data were integrated to generate a standardized gff3 file, and a high-resolution circular genomic map was rendered to visualize the findings.

## 3. Results and Analysis

### 3.1. Clinical Observation and Symptomatology of Diseased Turtles

Clinically, the afflicted tortoises presented with profound lethargy and a markedly diminished responsiveness to external stimuli. Respiratory distress was prominent, characterized by tachypnea and dyspnea, which manifested physically as frequent neck extensions and labored open-mouth breathing. These symptoms were accompanied by anorexia or a precipitous decline in nutritional intake. Gastrointestinal involvement was evidenced by diarrhea, the passage of mucoid or soft stools, and atypical darkening of fecal coloration. In severe instances, the clinical progression culminated in mortality.

### 3.2. Metagenomic Analysis of Symptomatic Geochelone sulcata

To comprehensively assess the potential pathogen landscape within the symptomatic *Geochelone sulcata* and circumvent the inherent limitations of traditional culture-based methods, we utilized metagenomic next-generation sequencing for unbiased pathogen screening of tissue specimens. The raw sequencing data underwent rigorous quality control (QC), which included the removal of adapter sequences, trimming of low-accuracy terminal bases, and the exclusion of reads containing over 20% ambiguous bases (N) or those with a length shorter than 20 bp. post-processing, the proportion of effective data reached 44.28%, with Q20 and Q30 scores of 97.88% and 94.55%, respectively, indicating high sequence fidelity (Table 2).

**Figure 1:**
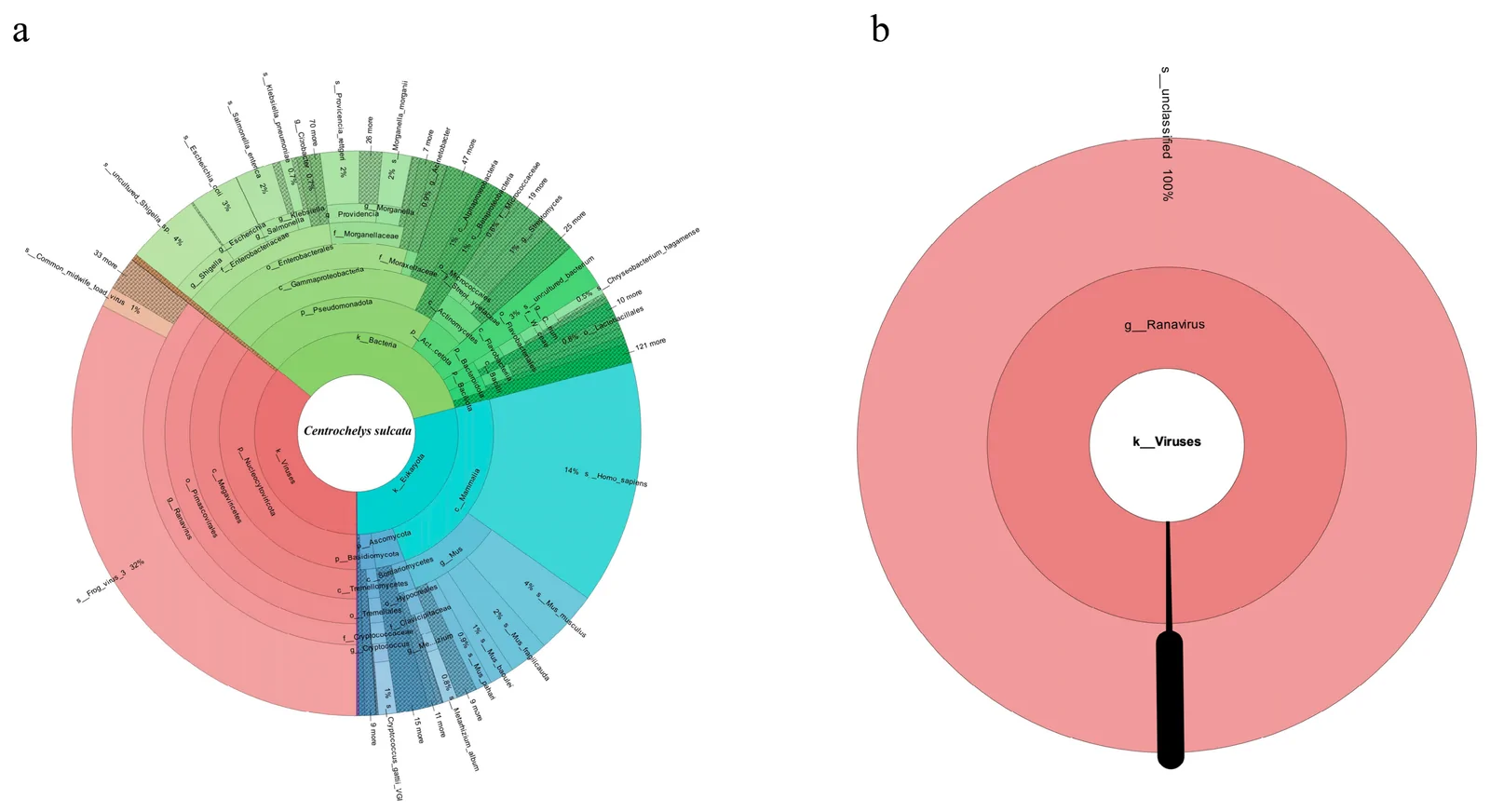
Metagenomic Sequencing Analysis in *Geochelone sulcata*. (**a**): Taxonomic distribution of metagenomic annotation results; (**b**): Visualization of viral abundance.

Upon aligning the clean reads against the NCBI viral database, viral taxa were found to account for 32% of the total reads. Notably, the genus *Ranavirus* constituted 32% of the viral fraction, identifying it as the predominant viral agent (Figure 1a). Subsequent de novo assembly of the QC-filtered data using SPAdes yielded 282,074 contigs, with a maximum contig length of 77,783 bp and an N50 value of 1315 bp (Table 3), reflecting robust assembly quality.

Gene annotation based on the assembled contigs identified seven putative viral sequences (Table S1). Of these, four were classified as ranavirus and three as Pea streak virus. Although Pea streak virus was successfully annotated at the contig level (TPM = 10.40), it lacked specific species-level nomenclature; furthermore, its sequences were characterized by short, fragmented distributions. Given the absence of prior reports regarding this virus in animal hosts, its presence was likely attributable to contamination from plant-based feed. In contrast, the ranavirus-related contigs exhibited greater length, high abundance (TPM = 2507.14), and complete genomic structural features, suggesting it as the primary etiological agent of the current outbreak. KRONA visualization of taxonomic abundance (Figure 1b) further confirmed the absolute dominance of ranavirus, while other viral taxa remained negligible.

### 3.3. Histopathological Characteristics in Geochelone sulcata

The macroscopic pathological observations were as follows: the pulmonary tissues exhibited marked swelling and a friable texture, with diffuse dark-red ecchymoses or petechial hemorrhages visible on both the serosal and cut surfaces; compression yielded a pale-red serosanguinous exudate. The liver was significantly enlarged with a tense capsule, displaying a pallid, grayish-yellow to ocher coloration. The pancreas was tumefied and fragile, presenting a grayish-red to dark-red hue. Renal involvement was characterized by organomegaly, a softened consistency, and a dark-red appearance, with an obscured corticomedullary junction and scattered brownish-yellow stippling. The intestinal mucosa exhibited congestion and edema, accompanied by focal or patchy mucosal necrosis and desquamation, resulting in superficial erosions or ulcer-like lesions.

**Figure 2:**
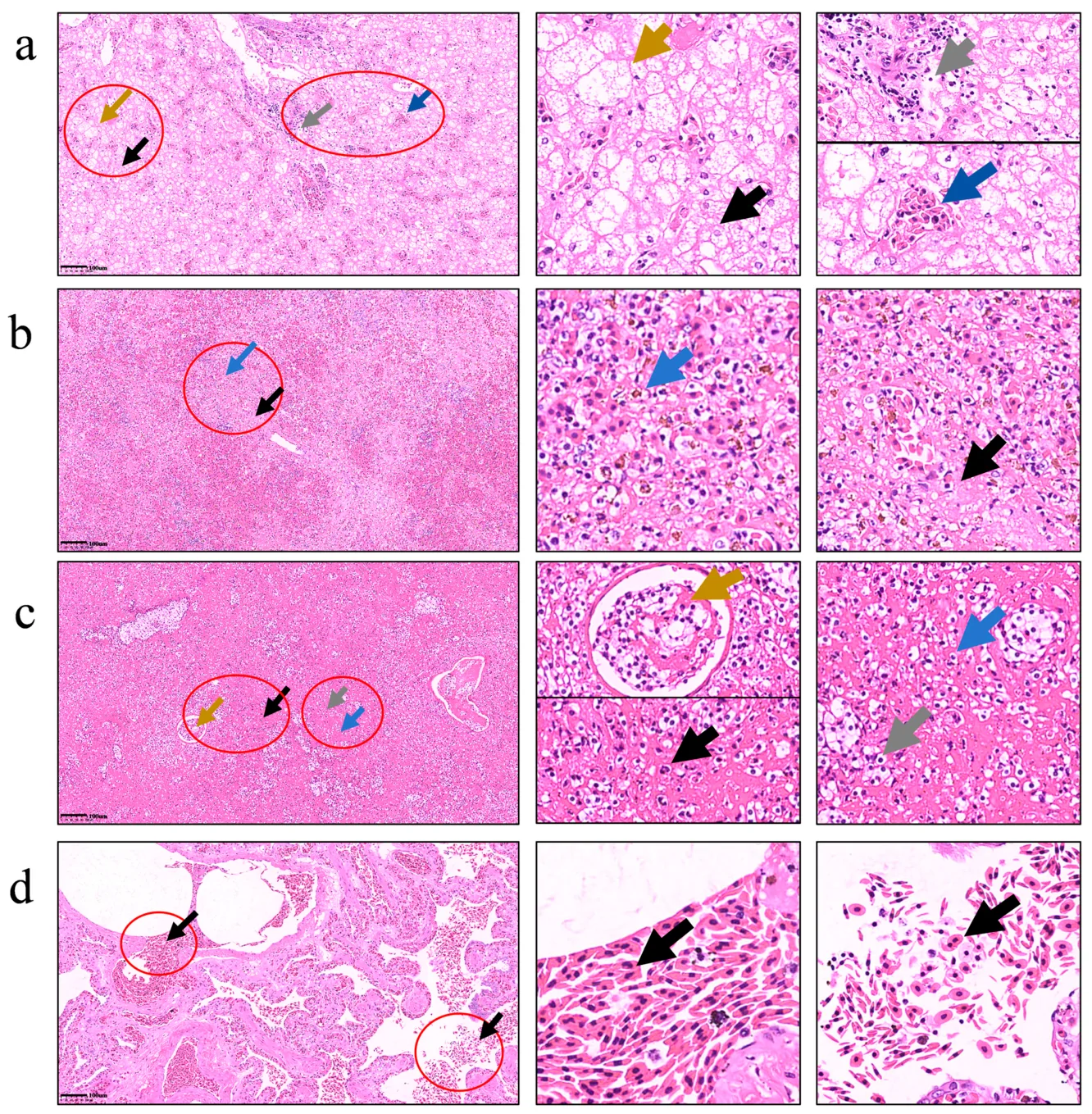
H&E Staining of Multiple Organs in Geochelone sulcata Exhibiting Acute Necrotic Lesions. (**a**) Liver: severe hepatocellular edema (black arrows), hepatocellular ballooning degeneration (golden arrows), sinusoidal congestion and dilation (blue arrows), scattered hepatocyte necrosis with neutrophil infiltration (gray arrows); (**b**) Spleen: white pulp necrosis and karyolysis (black arrows), intratissue lipofuscin deposition (blue arrows); (**c**) Pancreas: acinar cell necrosis (black arrows), necrotic islet cells with disordered architecture (golden arrows), lymphocytic infiltration (gray arrows), neutrophil infiltration (blue arrows); (**d**) Lung: intrabronchial haemorrhage (black arrows). Scale bars = 100 μm for all main panels; inset panels are 2× digitally magnified views of the circled lesion areas within the corresponding main panels.

To further elucidate the histopathological phenotype within the *Geochelone sulcata*, organ sections were subjected to H&E staining. The results revealed extensive acute injury, primarily characterized by parenchymal cell necrosis, vascular congestion, interstitial edema, and mixed inflammatory infiltration (Figure 2).

In the liver, severe hepatocellular edema, ballooning degeneration, and multifocal necrosis were observed, alongside significant sinusoidal congestion (Figure 2a). The spleen showed disintegration of the white pulp architecture, with widespread lymphocytolysis and karyorrhexis (Figure 2b). Within the pancreas, extensive acinar necrosis was noted, with the cytoplasm appearing as an eosinophilic homogeneous mass, accompanied by dense infiltration of lymphocytes and neutrophils (Figure 2c). Furthermore, the secondary bronchi of the lungs were engorged with erythrocytes, indicating acute hemorrhagic injury (Figure 2d). The multi-organ acute necrotizing lesions identified via histopathology are pathognomonic for viral infection. All diseased tortoises exhibited pathological lesions in target tissues, with upper respiratory tract symptoms observed in 100% of individuals. These findings are highly congruent with the high viral abundance detected through metagenomic sequencing in the same specimens, collectively implicating viral infection as the primary etiological factor of this outbreak.

### 3.4. Ultrastructural Characterization via Electron Microscopy of TFV

**Figure 3:**
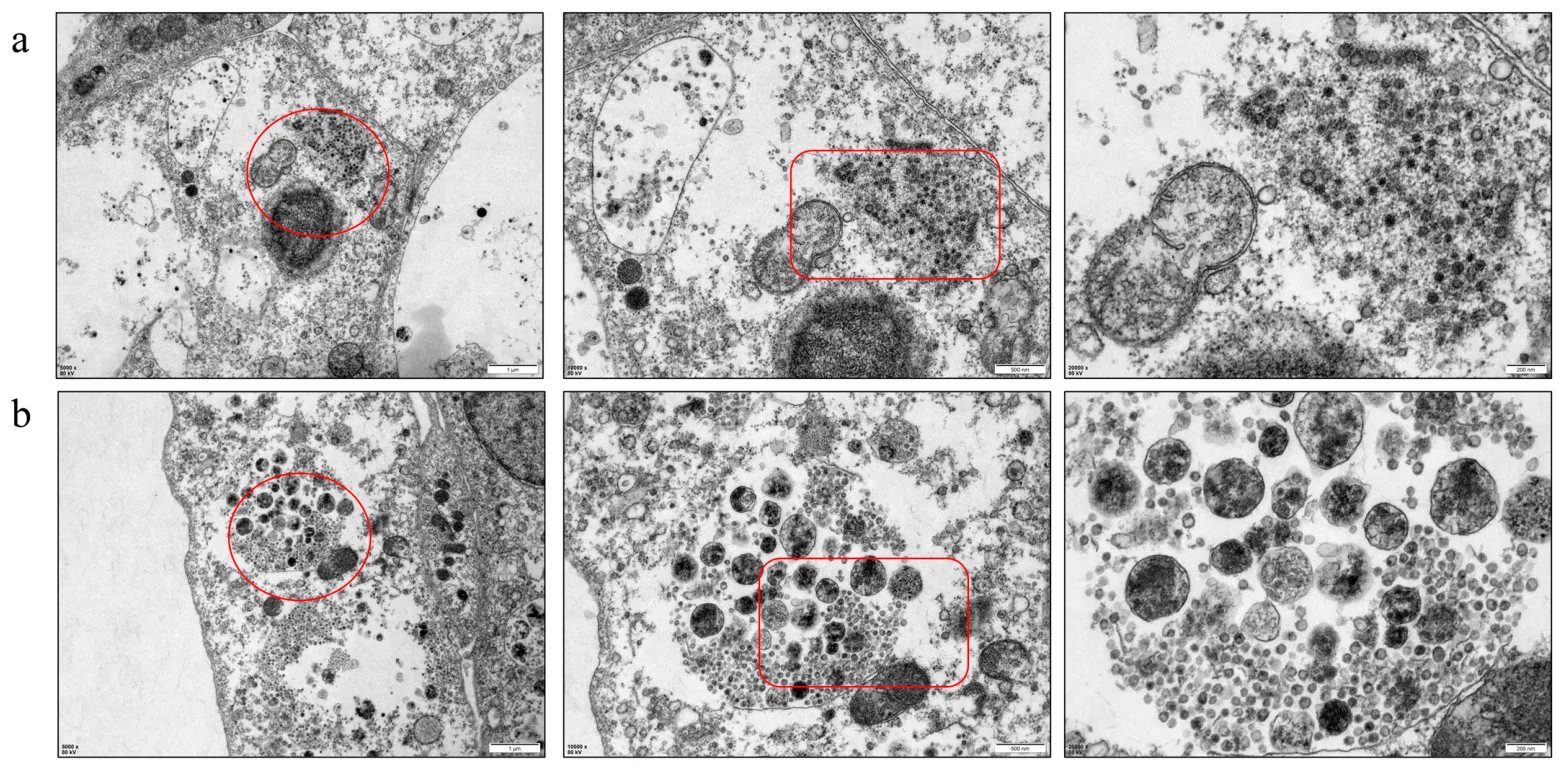
TEM Observation of Viral Infection in EPC Cells. (**a**,**b**): EPC cells at 48 h post-infection with the virus. Red annotated regions denote the aggregation of viral particles. Scale bars: 1 μm (**left panels**), 500 nm (**middle panels**), and 200 nm (**rightmost insets**). The micrographs were acquired at an accelerating voltage of 80 kV with a nominal magnification of ×10,000.

To further validate the presence of the virus and characterize its ultrastructural features, tissue homogenates from the symptomatic tortoises were inoculated onto EPC cells. TEM was performed 24 h post-infection. The results revealed that infected cells began to exhibit rounding, increased translucency, and shrinkage. As the CPE progressed, a multitude of spherical viral particles appeared within the infected cells. These particles have a measured apparent diameter of approximately 70 nm with a distinct electron-dense core, clustered into dense hexagonal viral aggregates localized at viral assembly sites (AS: specialized cytoplasmic subregions supporting virion construction and accumulation) (Figure 3). These tightly packed virion clusters build up inside assembly sites during particle maturation. Within the cytoplasm, vacuoles containing electron-dense granules were observed, associated with the clustering of viral particles (Figure 3a). Certain cells exhibited plasma membrane rupture with subsequent leakage of intracellular contents. Notably, the cytoplasm holds abundant circular cytoplasmic inclusion bodies (Figure 3b), with viral particles densely packed inside these compartments. Such electron-dense inclusion bodies are membrane-free cytoplasmic compartments dedicated to viral genome replication and storage of newly produced virions. The prominent large circular ultrastructures inside assembly sites in Figure 3b cannot be conclusively identified with existing experimental evidence. Repeated sectioning excludes routine TEM processing artifacts; comparable morphologies are rarely documented across published ranavirus TEM datasets from EPC cell infection. These compartments serve as core intracellular hubs for viral replication and particle assembly. Collectively, these TEM observations confirm abundant clustered virus-like particles alongside widespread cellular structural damage. Repeated manual sizing across multiple micrographs yields a consistent apparent virion diameter of 70 nm. The reduced measured value relative to the canonical 150–170 nm ranavirus dimension can be explained by tangential non-equatorial ultrathin sectioning, partial dehydration shrinkage during TEM sample preparation, or measurement targeted at the inner electron-dense viral core. This indicates robust intracellular viral proliferation; host cell lysis facilitates virion release and environmental dissemination.

### 3.5. Molecular Detection and Phylogenetic Analysis of TFV

Transmission electron microscopy revealed the presence of characteristic iridovirus-like particles within infected EPC cells, identified by pathognomonic features such as enveloped virions, hexagonal crystalline arrays, and intracytoplasmic inclusion bodies. To further corroborate the identity of the pathogen at the molecular level, specific primers were designed based on the MCP gene sequences of ranavirus available in the NCBI database. PCR amplification was subsequently performed on the cell culture samples inoculated with tissue homogenates from the diseased tortoises.

**Figure 4:**
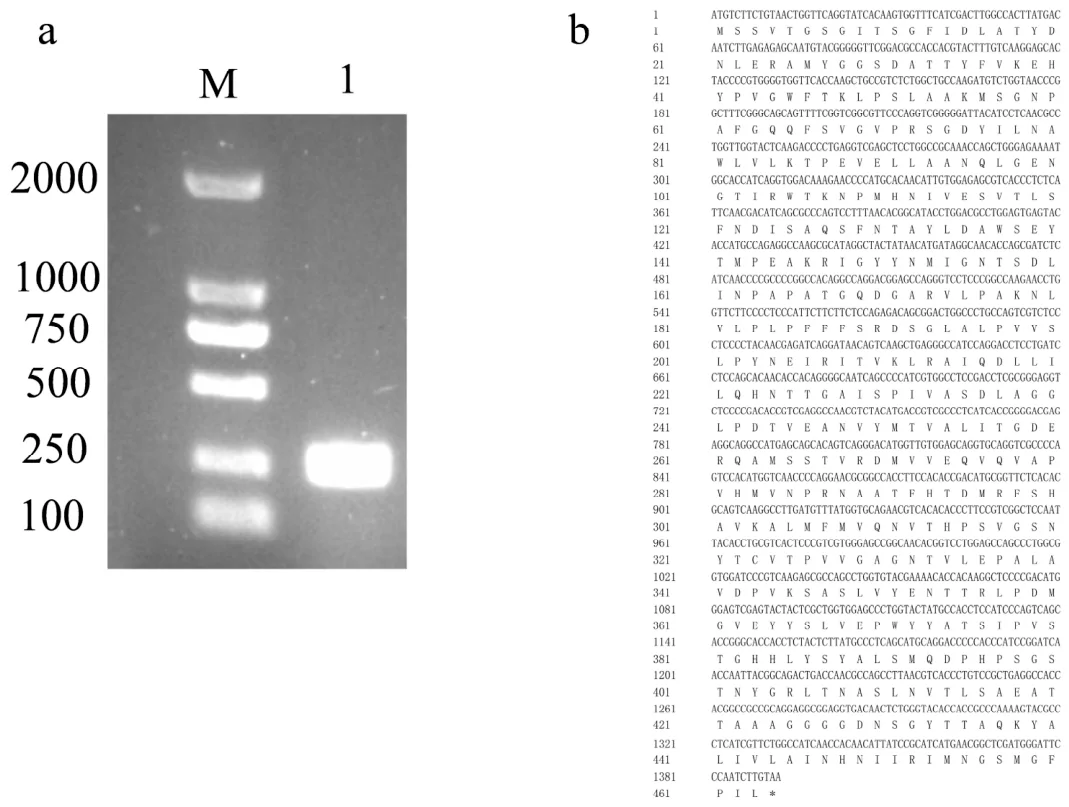
Detection of the TFV MCP Gene and the Full-Length Sequence of the ORF Region. (**a**): PCR-based detection in EPC cell samples; (**b**): ORF sequence of the MCP gene of the TFV-CN2025 strain and its deduced amino acid sequence. M: DL2000 DNA molecular weight marker; 1: EPC cell sample; asterisks (*) denote stop codons.

**Figure 5:**
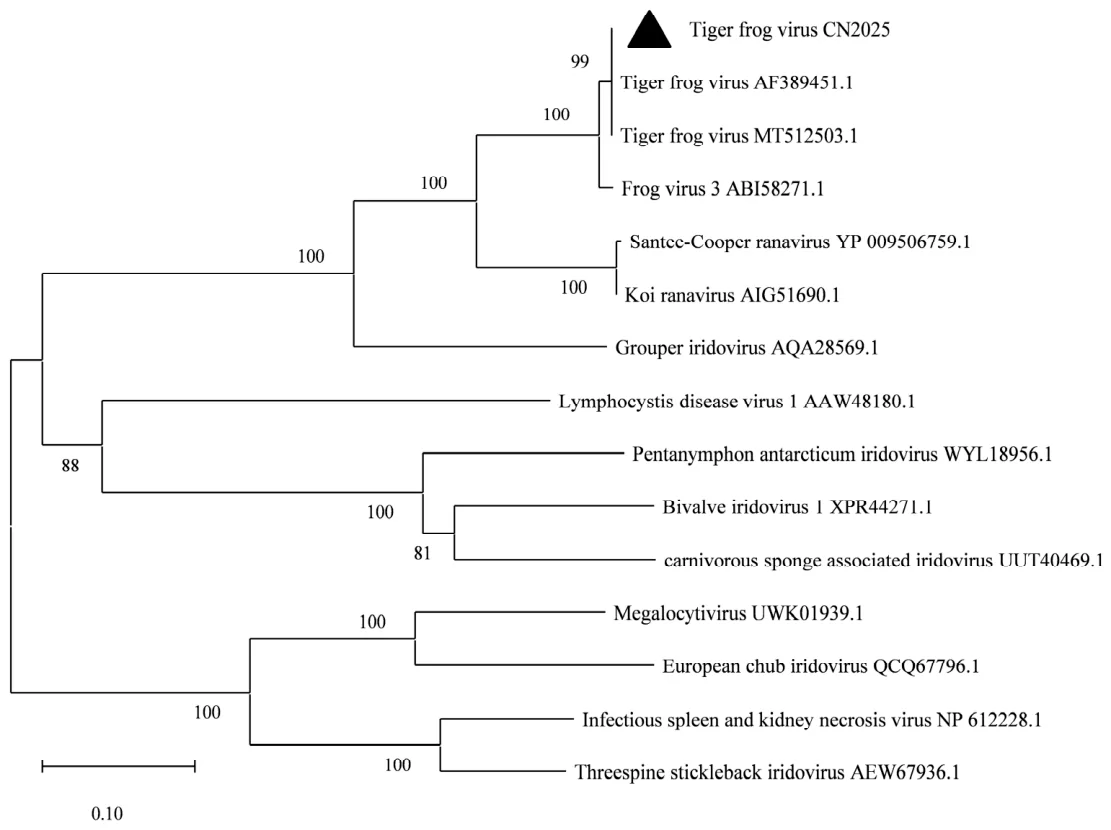
Phylogenetic Tree Constructed Based on MCP Amino Acid Sequences. Node values mean bootstrap supports (1000 replicates). The bootstrap value of 99 for the strain CN2025 branch reveals high topological reliability. The triangle marks the isolate from this study, and the scale bar shows a genetic distance of 0.10.

The analysis yielded a single, distinct positive band at 260 bp (Figure 4a). Following the cloning and sequencing of the PCR products, the full-length sequence of the MCP gene’s Open Reading Frame (ORF) and its deduced amino acid sequence were obtained (Figure 4b). Phylogenetic analysis was conducted using MEGA software, comparing the MCP sequence of the isolate with those of 14 other iridovirus strains. The resulting phylogeny (Figure 5) demonstrated that the amino acid sequence of the isolated virus clustered within the genus *Ranavirus*. These molecular findings confirm that the pathogen belongs to the genus *Ranavirus* within the family Iridoviridae; consequently, the isolate has been designated as the TFV strain TFV-CN2025.

### 3.6. Whole-Genome Sequencing and Genomic Characterization of TFV

Following the successful isolation and identification of the TFV-CN2025, we performed whole-genome sequencing and assembly to further elucidate its genetic architecture and molecular characteristics. The raw sequencing data were subjected to rigorous quality control (QC) protocols, including the removal of adapter sequences, trimming of low-quality terminal bases, and the exclusion of reads with an ambiguous base (N) ratio exceeding 20% or a length under 20 bp. post-QC analysis yielded Q20 and Q30 scores of 99.35% and 97.67%, respectively (Table 4), reflecting superior data fidelity (Figure S1) suitable for high-resolution genomic assembly.

Using the SPAdes assembly algorithm, a complete linear genomic sequence of 104,328 bp was reconstructed from a single contig, with both the N50 value and maximum contig length reaching 104,328 bp (Table 5). The assembled sequence exhibited no base gaps (N) and a balanced GC content of 55.13% (Table 6), underscoring the structural integrity and reliability of the assembly.

To validate the assembly, clean reads were re-mapped to the consensus genome. This revealed an average sequencing depth of 7933.04× (Table 7), with uniform coverage across the entire sequence and an absence of significant gaps or aberrant peaks (Figure S1). These metrics further substantiate the accuracy and completeness of the genomic reconstruction.

Subsequent structural prediction and functional annotation identified 95 ORF (Table 8), with coding sequence lengths predominantly clustered between 100 and 400 amino acids (Figure S1). A high frequency of G/C-terminated codons was observed—such as Gly-GGC (22.21%) and Asp-GAC (53.29%)—aligning with the overall GC content of 55.13% and indicating a pronounced GC bias (Table 9). Functional annotation revealed that 97.89% of the 95 encoded proteins were successfully annotated in at least one database (Table 10), with the highest coverage found in RefSeq (97.89%) and SwissProt (82.11%).

**Figure 6:**
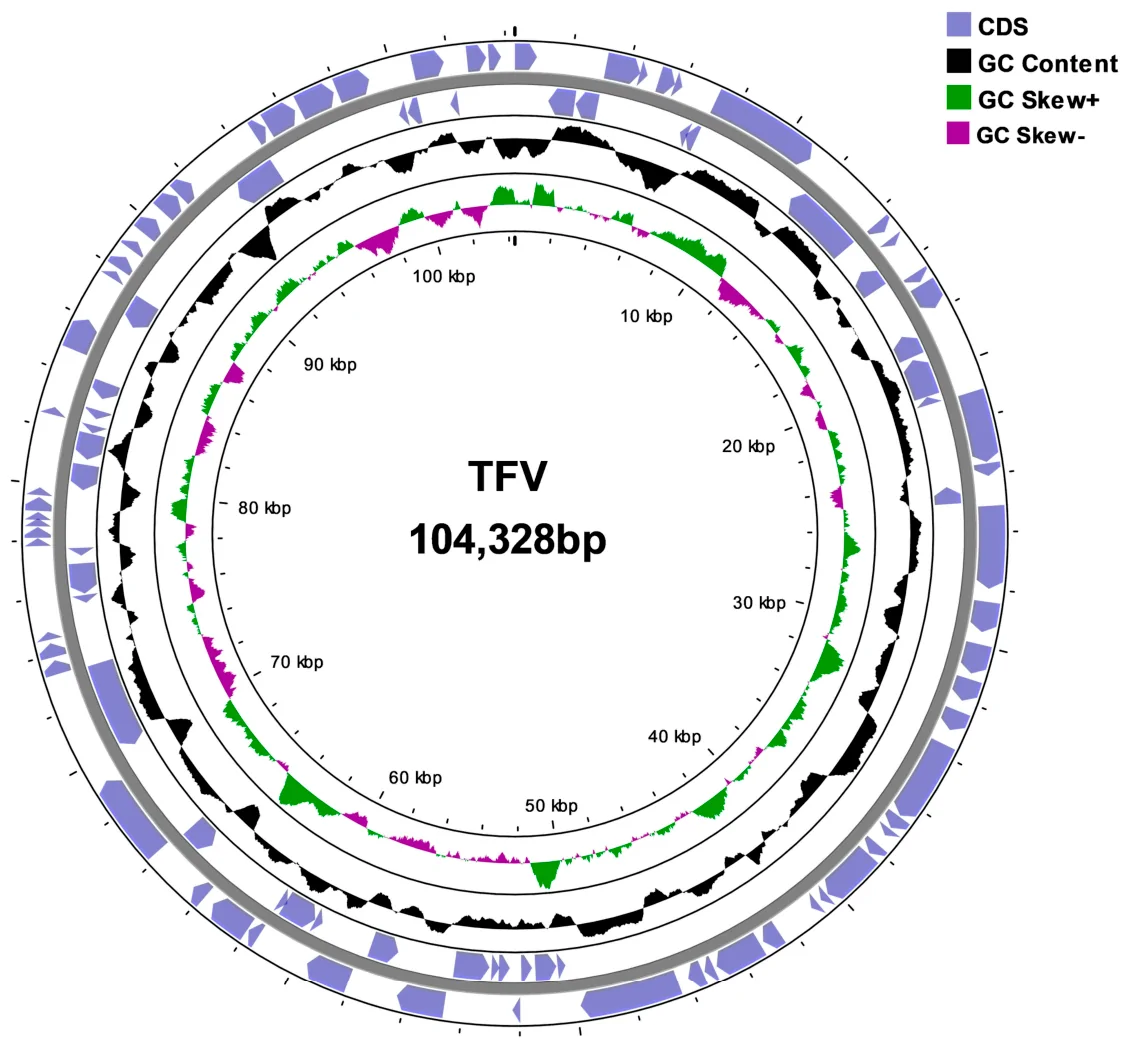
Circular Map of the Genome. Note: CDS, coding sequence; GC Skew+, GC skew of the positive strand; GC Skew-, GC skew of the negative strand.

BLAST analysis demonstrated 99.8% nucleotide identity between the isolate and the TFV reference sequence, confirming that TFV-CN2025 represents a full-length TFV genome. Comprehensive analysis suggests that the genome possesses characteristic features of small double-stranded DNA (dsDNA) viruses, including widely distributed coding sequences (CDS) on both strands and a uniform GC distribution. Furthermore, GC-skew analysis highlighted potential origins of replication (Figure 6). Distinct shifts in GC-skew values mark genomic positional transitions linked to the onset of bidirectional genome replication. Collectively, these findings define the genomic composition of TFV-CN2025 and provide a critical foundation for future studies on its phylogeny and functional genomics.

The raw sequence data reported in this paper have been deposited in the Genome Sequence Archive (Genomics, Proteomics & Bioinformatics 2025) [16] in National Genomics Data Center (Nucleic Acids Res 2025) [17], China National Center for Bioinformation/Beijing Institute of Genomics, Chinese Academy of Sciences (GSA Accession No.: CRA044438) that are publicly accessible at https://ngdc.cncb.ac.cn/gsa (accessed on 10 June 2026).

## 4. Discussion

In recent years, artificial breeding of *Geochelone sulcata* has developed into a large-scale industry [18]. Given its market value exceeding 3000 RMB per individual, this species has become a prominent ornamental tortoise widely farmed in southern China. Despite its considerable economic importance, systematic investigations into its infectious diseases and associated pathogens remain severely limited worldwide. Ranaviruses are listed as notifiable animal diseases by the World Organisation for Animal Health (WOAH) and can infect a broad spectrum of ectothermic vertebrates, including fish, amphibians, and reptiles. Infections caused by ranaviruses often lead to acute systemic diseases with high mortality, and these pathogens are recognized as a major factor driving global amphibian population decline [19]. Accumulated evidence has proven that the host range of ranaviruses is continuously expanding. To date, ranavirus outbreaks have been reported in at least 25 countries, and these viruses are considered a potential threat to approximately half of the affected wildlife species [20]. Previous studies have confirmed that *Ranavirus rana1* can cause lethal infections in tortoises [21]. A large-scale mortality event caused by an *Ranavirus rana1*-like virus was also documented in box turtles in Maryland, USA [22], further demonstrating the strong cross-species transmission potential of ranaviruses among different reptilian hosts. Nevertheless, epidemiological data and detailed pathological analyses of reptile-associated ranaviruses are still scarce. The recent mass mortality of more than twenty *G. sulcata* individuals not only resulted in substantial economic losses but also posed a severe threat to the sustainable development of the tortoise breeding industry. In the present study, we combined metagenomic sequencing and conventional virus isolation techniques and successfully isolated Tiger Frog Virus (TFV) from diseased *G. sulcata* for the first time globally. Multiple analytical approaches were further applied to clarify the taxonomic status of this viral strain within the genus *Ranavirus*.

Histopathological examination revealed typical lesions in multiple visceral organs of infected *G. sulcata*, including pulmonary hemorrhage, hepatocellular ballooning degeneration, splenic necrosis, renal tubular edema, pancreatic acinar necrosis, and intestinal mucosal injury. These extensive pathological changes in parenchymal organs indicated severe systemic infection and multiple organ dysfunction syndrome (MODS), which reflected the strong systemic invasiveness of the isolated TFV strain. The histopathological characteristics and acute lethal symptoms observed in this study were highly consistent with systemic necrotizing inflammation caused by *Ranavirus rana1* in three-toed box turtles (*Terrapene carolina triunguis*) reported by Apakupakul et al. [11]. The similarity in disease progression further verifies the high pathogenicity of ranaviruses to terrestrial chelonians and suggests that the clinicopathological features of ranavirus infection are relatively conserved across different chelonian species. Our results are also in accordance with the multi-organ necrosis caused by *Ranavirus rana1*-like viruses in juvenile Meller’s chameleons (*Trioceros melleri*) [23]. Lizard infections with ranaviruses also present high lethality and similar clinical signs including skin lesions [24]. For reptile hosts, TFV infection typically triggers systemic visceral hemorrhage and multi-organ necrotic lesions, whereas *Ranavirus rana1* tends to induce prominent epidermal ulceration alongside milder internal tissue necrosis. Moderate inter-strain pathogenic variation may stem from host-specific viral adaptive traits. All these findings collectively prove that ranaviruses possess broad host adaptability and strong virulence in terrestrial reptiles, with shared pathological characteristics among infected hosts. Notably, splenic necrosis can be regarded as a key histopathological indicator for the diagnosis of ranavirus infection in chelonians.

Ranaviruses are mainly transmitted among ectothermic vertebrates via contaminated water, fomites, and direct contact [25]. Mosquitoes have also been proposed as potential mechanical vectors for ranavirus transmission among tortoises [26], and vertical transmission from parent to offspring has also been suspected [27]. Environmental temperature is another critical factor affecting disease progression: low temperature can reduce circulatory function and phagocytic activity, thereby lowering the risk of systemic infection. Phagocytes are proven to be the key cells mediating the in vivo spread of *Ranavirus rana1* [28], with this core conclusion first experimentally validated by Robert and collaborators in earlier published work [29]. Given their broad host range, high virulence, and diverse transmission routes, coupled with the frequent cross-regional trade of amphibians, fish, and reptiles, certain ranavirus strains pose a serious threat to wild animal populations and may even lead to local species extinction [25]. Under the mixed captive rearing setup at this facility, shared circulating water and direct interspecies contact are judged as the most plausible transmission drivers fueling this TFV outbreak. Considering the current lack of systematic pathological data on TFV infection in *G. sulcata*, the present pathological findings provide a solid morphological basis for the clinical diagnosis and differential detection of this disease.

TFV exhibits a wide cellular tropism and can infect multiple fish cell lines, including grass carp ovary (CO) cells, epithelioma papulosum cyprini (EPC) cells, fathead minnow muscle cells, mandarin fish fry cell-1 (MFF-1) and zebrafish embryo fibroblast (ZF4) cells [30]. Moreover, TFV can also infect mammalian cell lines such as human hepatocellular carcinoma (HepG2) cells [31]. In this study, homogenates of diseased tissue were inoculated into EPC cells for virus propagation. Electron microscopy ultrastructural observation revealed massive viral particles proliferating in the cytoplasm of infected cells, which presented regular hexagonal morphology with a diameter of approximately 70 nm. Some previous studies reported the virion diameter of related ranaviruses to be around 150 nm [32]. We speculate that the discrepancy in virion size is mainly attributed to differences in host cell types or viral subtypes, as host factors can affect viral morphogenesis [33]. Reported TEM outcomes from previously isolated TFV strains consistently yield capsid diameters above 100 nm under equivalent EPC cell culture conditions, ruling out inherent inter-strain structural divergence as the main driver. The 70 nm readings most likely stem from partial tangential sectioning of spherical virions during ultramicrotomy: cutting through the periphery of a round particle naturally produces a smaller cross-sectional width than the true full particle diameter. Whole-genome sequencing was further performed to characterize the pathogen, and the results confirmed that the viral genome is double-stranded DNA (dsDNA). Homology searches against the NCBI database verified that the isolated strain belongs to the genus *Ranavirus* in the family Iridoviridae, and was definitively identified as TFV.

The major capsid protein (MCP) is the main structural protein composing the icosahedral capsid of iridoviruses. Due to its high sequence conservation within the genus *Ranavirus*, the MCP gene has become a classic molecular marker for species identification and phylogenetic analysis of ranaviruses [34]. In the present work, based on the metagenomic sequencing data obtained from infected *G. sulcata*, we combined PCR amplification and successfully amplified a 260 bp fragment of the MCP gene. BLAST analysis confirmed the integrity of its open reading frame (ORF), and the sequence shared 99% identity with the published sequences of TFV and *Ranavirus rana1*. Phylogenetic analysis based on the full-length MCP gene showed that the isolated strain, designated TFV-CN2025, clustered within the TFV lineage of the *Ranavirus rana1*-like group and formed a tight clade with classical *Ranavirus rana1* strains, which further supported its taxonomic classification within the genus *Ranavirus*.

Although phylogenetic analysis based on the MCP gene provides a preliminary taxonomic framework for TFV-CN2025, relying exclusively on highly conserved core genes is manifestly insufficient to elucidate the mechanisms underlying the cross-species spillover of this virus from amphibians to reptiles. In this context, comparative whole-genomics holds indispensable value for deciphering its potential for interspecies transmission. Consequently, we further conducted whole-genome sequencing and assembly of the isolate (The data reported in this paper have been deposited in the GenBase [35] in National Genomics Data Center [36], Beijing Institute of Genomics, Chinese Academy of Sciences/China National Center for Bioinformation, under accession number C_AA474083.1 that is publicly accessible at https://ngdc.cncb.ac.cn/genbase (accessed on 10 June 2026)).

Alignment analyses revealed that the complete genome of TFV-CN2025 shares a striking nucleotide homology of 99.8% with established TFV reference sequences. Furthermore, 95 ORFs were predicted within the genome, of which 97.89% were assigned definitive functional annotations in current databases, with the primary coding regions concentrated within the 100–400 aa range. Nevertheless, the precise fine structures of these 95 ORFs, along with the non-core gene variations they represent, remain critical determinants of the virus’s pathogenic properties. Logan [6], in a whole-genome comparative study of wood frog-derived *Ranavirus rana1* isolates, noted that even when divergent ranavirus isolates exhibit exceptionally high whole-genome nucleotide similarity (>99%), insertions, deletions, or unique recombinations within non-core gene regions can still substantially alter viral nucleic acid metabolism, immune evasion capabilities, and host tropism. This perspective is particularly consequential in the context of cross-taxa transmission. Similarly, Kim [5], in reporting endemic *Ranavirus rana1*-like viruses from wild Korean clawed salamanders in Asia, confirmed a marked decline in similarity (approximately 84.2%) at the level of specific ORFs between these amphibian-derived viruses and certain reptile-derived iridoviruses. This strongly indicates that ranaviruses undergo host-adaptive genomic differentiation during evolutionary succession. Consequently, the diverse proteins encoded within the 100–400 aa range of TFV-CN2025—extending beyond conserved core proteins such as the MCP and DNA polymerase—along with certain specific ORFs or hypothetical proteins it harbors, are highly likely to constitute the pivotal molecular genetic basis enabling this virus to breach the amphibian host barrier, successfully establish infection, and induce pathogenesis within reptilian hosts.

According to the International Committee on Taxonomy of Viruses (ICTV), seven species are currently recognized within the genus *Ranavirus*, namely *Ranavirus rana1*, Common midwife toad virus (CMTV), Ambystoma tigrinum virus (ATV), European North Atlantic Ranavirus (ENARV), Epizootic hematopoietic necrosis virus (EHNV), Santee-Cooper Ranavirus (SCRV) and Singapore grouper iridovirus (SGIV). *Ranavirus rana1* -like viruses have an extremely broad host range and can infect amphibians, fish, and reptiles [12]. *Ranavirus rana1* was first isolated from *Lithobates pipiens* in the United States [37]. Genomic analysis indicated that the *Ranavirus rana1* strain infecting Meller’s chameleons shares high homology with the virus isolated from eastern box turtles [9]. Whether the differences in viral pathogenicity are associated with host range expansion or adaptive infection to different developmental stages of the same host remains to be further explored.

The revised 2026 Catalogue of Animal Pathogenic Microorganisms issued by the Ministry of Agriculture and Rural Affairs of China has officially incorporated pathogens associated with exotic pets and chelonians into the national biosafety management system. Ranaviruses possess strong cross-species transmission capacity, environmental tolerance, and high lethality, which pose substantial risks to the sustainable development of China’s exotic pet industry, the stability of zoo animal populations and national ecological security.

This study is the first report on the isolation and comprehensive characterization of TFV in captive *G. sulcata* in China. We confirmed that TFV can cause acute multi-organ necrotic lesions with high pathogenicity in *G. sulcata*. Genomic and phylogenetic analyses demonstrated that TFV-CN2025 belongs to the *Ranavirus rana1* lineage of the genus *Ranavirus* and shares high sequence homology with amphibian-derived ranaviruses. All results clearly prove that ranaviruses have successfully achieved cross-species transmission from amphibian hosts to terrestrial tortoises.

In view of the above findings, we strongly recommend including ranavirus detection in the routine pathogen screening and quarantine work for tortoises and other exotic reptiles. Strengthened biosafety supervision should be implemented in commercial breeding farms, zoos, and wildlife rescue stations, to prevent ranavirus cross-species transmission and large-scale disease outbreaks.

## Figures and Tables

**Table 1:** PCR Amplification Protocol and Reaction System.

Component	Volume (μL)
rTaq	12.5
DNA Template	1
Forward Primer	1
Reverse Primer	1
ddH_2_O	9.5
Total Volume	25

**Table 2:** Summary of Metagenomic Sequencing Data Preprocessing Results.

Sample	Total Reads	Total Bases	Clean Reads	Clean Bases	Q20 Rate (%)	Q30 Rate (%)	GC (%)	Nohost Reads	Efficient (%)
G0608	281,367,256	42,205,088,400	279,179,440	39,951,480,382	97.98	94.55	49.54	123,635,880	0.29

**Table 3:** Statistics of De Novo Assembly Results.

Sample	Genome Size (bp)	Contig Number	Longest Contig (bp)	Contig N50 (bp)	Average Contig (bp)	Shortest Contig (bp)
G0608	337,893,869	282,074	77,783	1315	1197.89	501

**Table 4:** Statistics of Data Throughput and YieldTable.

Sample	Total Reads	Total Bases	Clean Reads	Clean Bases	Q20 Rate (%)	Q30 Rate (%)	GC (%)
Tfv	80,809,484	12,121,422,600	79,910,762	10,684,814,644	99.35	97.67	45.68

**Table 5:** Comprehensive Statistics of Assembly Metrics.

Genome Size (bp)	Contig Number	Contig N50 (bp)	Longest Contig (bp)	Shortest Contig (bp)
104,328	1	104,328	104,328	104,328

**Table 6:** Statistics of Genomic Nucleotide Distribution.

Base	Number (bp)	% Genome
A	23,932	22.94
T	22,883	21.93
G	29,202	27.99
C	28,311	27.14
N	0	0.00
GC	57,513	55.13
Total	104,328	100.00

**Table 7:** Statistics of Genomic Base Composition and Sequencing Depth.

Contig	Length (bp)	GC Content (%)	Sequencing Depth (X)	Topology
tig00001	104,328	55.13	7933.0400	linear

**Table 8:** Statistics of Genomic Structural Prediction.

Type	Number	Length (bp)	% Genome
tRNA	0	0	0
CDS	95	82,527	79.10
CRISPR	0	0	0
genomic_island	0	0	0
prophage_region	0	0	0

**Table 9:** Codon Usage Bias Table.

Amino Acid	Codon	Number	Frequency (1/1000)
Gly	GGG	514	18.68
Gly	GGA	570	20.72
Gly	GGT	276	10.03
Gly	GGC	611	22.21
Glu	GAG	1276	46.38
Glu	GAA	297	10.80
Asp	GAT	249	9.05
Asp	GAC	1466	53.29

**Table 10:** Functional Annotation Statistics of Genome-Encoded Proteins.

Database	Annotated Number	% All
COG	9	9.47
KEGG	11	11.58
GO	20	21.05
Refseq	93	97.89
Pfam	41	43.16
TIGRFAMs	3	3.16
SwissProt	78	82.11
all databases	2	2.11
at least one database	93	97.89
Overall	95	100

## Data Availability

All data generated in this study were submitted to the Genome Sequence Archive (GSA). Access links for these datasets are available in the Results Section 3.

## Supplementary Materials

The following supporting information can be downloaded at: https://www.mdpi.com/article/10.3390/v18080810/s1, Figure S1: Data Statistics and Quality Control Profiles; Table S1: Taxonomic Classification and Statistics of Viral Sequences.
