# Supplementary material for: Cross-Species Spillover of Tiger Frog Virus Caused Lethal Systemic Disease in Captive Geochelone sulcata: Etiology, Pathology, and Genomic Characterization
Source: Viruses. 2026 Jul 23;18(8):810. doi: 10.3390/v18080810 (PMC13517490; doi:10.3390/v18080810)
Supplement: Supplementary file 1 [file viruses-18-00810-s001.zip › viruses-4430533-supplementary.pdf]

Table S1. Taxonomic Classification and Statistics of Viral Sequences.

Kingdom	Phylum	Class	Order	Famil	Genus	Species	Contigs Number	Contigs Length (bp)	TPM
Viruses	Nucleocytoviricota	Megaviricetes	Pimascovirales	Iridoviridae	Ranavirus	no support	4	101,860	2507.14
Viruses	Kitrinoviricota	Alsuviricetes	Tymovirales	Betaflexiviridae	Carlavirus	Pea Streak virus	3	4,948	10.40

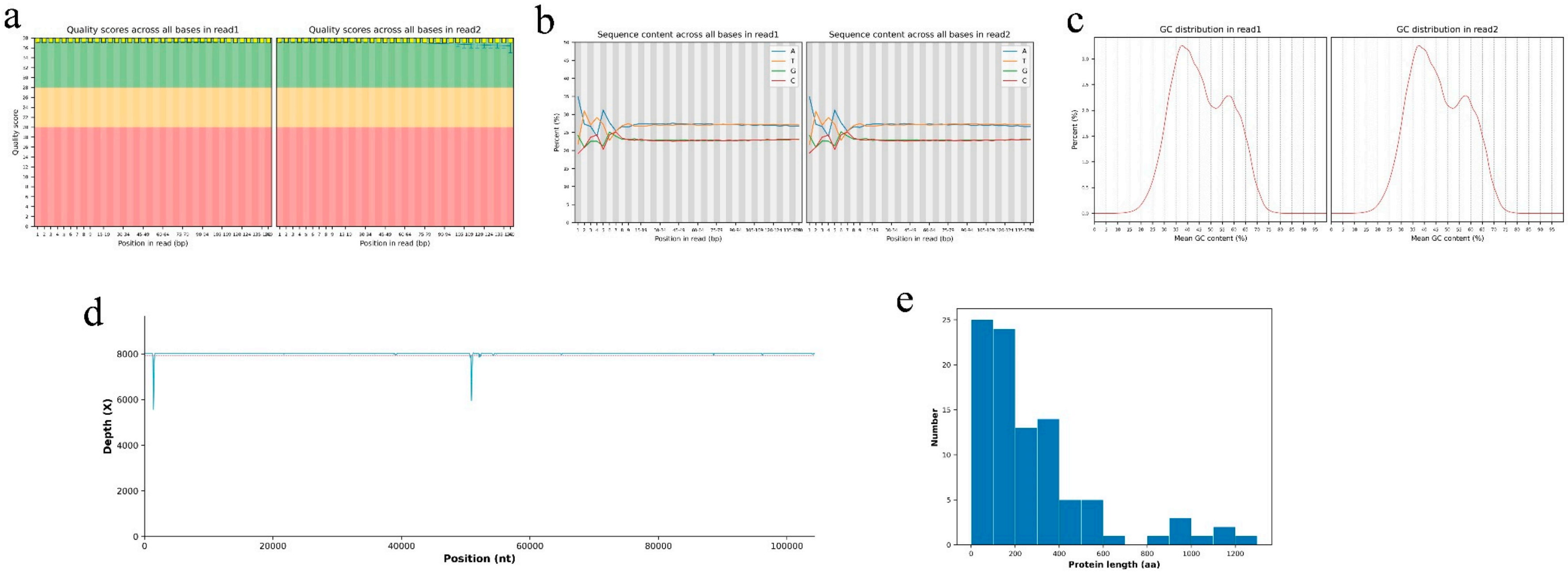

Figure S1. Data Statistics and Quality Control Profiles.
